# Aerobic-anaerobic transition boosts poly(3-hydroxybutyrate-co-3-hydroxyvalerate) synthesis in *Rhodospirillum rubrum*: the key role of carbon dioxide

**DOI:** 10.1186/s12934-023-02045-x

**Published:** 2023-03-10

**Authors:** Manuel S. Godoy, Santiago R. de Miguel, M. Auxiliadora Prieto

**Affiliations:** 1 Polymer Biotechnology Lab, Biological Research Centre Margarita Salas, Spanish National Research Council (CIB-CSIC), Madrid, Spain; 2 Interdisciplinary Platform for Sustainable Plastics towards a Circular Economy‐CSIC (SusPlast‐CSIC), Madrid, Spain

**Keywords:** *Rhodospirillum rubrum*, Poly(3-hydroxybutyrate-*co*-3-hydroxyvalerate), PHBV, Purple nonsulfur bacteria, Calvin-Benson-Bassham cycle, Electron sink

## Abstract

**Background:**

Microbially produced bioplastics are specially promising materials since they can be naturally synthesized and degraded, making its end-of-life management more amenable to the environment. A prominent example of these new materials are polyhydroxyalkanoates. These polyesters serve manly as carbon and energy storage and increase the resistance to stress. Their synthesis can also work as an electron sink for the regeneration of oxidized cofactors. In terms of biotechnological applications, the co-polymer poly(3-hydroxybutyrate-*co*-3-hydroxyvalerate), or PHBV, has interesting biotechnological properties due to its lower stiffness and fragility compared to the homopolymer poly(3-hydroxybutyrate) (P3HB). In this work, we explored the potentiality of *Rhodospirillum rubrum* as a producer of this co-polymer, exploiting its metabolic versatility when grown in different aeration conditions and photoheterotrophically.

**Results:**

When shaken flasks experiments were carried out with limited aeration using fructose as carbon source, PHBV production was triggered reaching 29 ± 2% CDW of polymer accumulation with a 75 ± 1%mol of 3-hydroxyvalerate (3HV) (condition C2). Propionate and acetate were secreted in this condition. The synthesis of PHBV was exclusively carried out by the PHA synthase PhaC2. Interestingly, transcription of *cbbM* coding RuBisCO, the key enzyme of the Calvin-Benson-Bassham cycle, was similar in aerobic and microaerobic/anaerobic cultures. The maximal PHBV yield (81% CDW with 86%mol 3HV) was achieved when cells were transferred from aerobic to anaerobic conditions and controlling the CO_2_ concentration by adding bicarbonate to the culture. In these conditions, the cells behaved like resting cells, since polymer accumulation prevailed over residual biomass formation. In the absence of bicarbonate, cells could not adapt to an anaerobic environment in the studied lapse.

**Conclusions:**

We found that two-phase growth (aerobic-anaerobic) significantly improved the previous report of PHBV production in purple nonsulfur bacteria, maximizing the polymer accumulation at the expense of other components of the biomass. The presence of CO_2_ is key in this process demonstrating the involvement of the Calvin-Benson-Bassham in the adaptation to changes in oxygen availability. These results stand *R. rubrum* as a promising producer of high-3HV-content PHBV co-polymer from fructose, a PHBV unrelated carbon source.

**Supplementary Information:**

The online version contains supplementary material available at 10.1186/s12934-023-02045-x.

## Introduction

Bio-based materials are one of the main hopes in the industry for the substitution of most types of plastics in a sustainable strategy. Industrial production and commercialization of bioplastics started over 30 years ago but only in the last years the market demand for sustainable materials and products has been on the rise mainly due to a global awareness on plastic pollution and climate change [1]. Within this group, microbially produced bioplastics are specially promising materials since they can be naturally degraded, making its end-of-life management more amenable with the environment [2, 3]. This scenario brings a new biotechnological challenge: how to make these polymers more suitable for applications so far fulfilled by conventional plastics. Properties such as elasticity, temperature resistance, water and gas permeability, etc., defines the application that the material may accomplish [4].

Structurally, PHA are linear polymers of hydroxy acids that are connected by an ester bond. In the intracellular milieu, the polyester chains form granules associated to proteins (GAPs), in a way that they can dynamically interact with the intracellular processes. Accumulated as a response to unbalanced nutrient conditions, generally more carbon than nitrogen or phosphate, PHA becomes a drift to which the cell may resort to in case of energy or carbon scarcity [5, 6, 8]. The PHA synthases are the enzymes responsible for the polymerization of this carbon units, by transforming (*R*)-3-hydroxyacyl-CoA units into a polyester chain and releasing one CoA molecule per catalytic cycle [9].

PHA properties are determined mainly by the monomer composition. When different types of monomers are combined in the same chain, the final heteropolymers present different characteristics respect to the corresponding homopolymers. For example, the extracted PHB becomes highly crystalline, which results into a stiff but brittle material. Furthermore, the ability to process the homopolymer is limited by the melting point (around 170 °C) which is close to the decomposition temperature. Combining 3HB with 3-hydroxyvalerate (3HV) in the same polymer, gives the resulting poly(3-hydroxybutyrate-*co*-3-hydroxyvalerate) (PHBV) co-polymer more stress-resistant properties by reducing the crystallinity. The difficulty to arrange in ordered microscopic structures decreases its stiffness and fragility compared to PHB. A recent review explaining the advantages of heteropolymers over homopolymer has recently been published [10]. PHBV can form films with excellent gas and water barrier properties, similar to those of polypropylene, without losing other convenient mechanical properties of PHB [11].

**Fig. 1:**
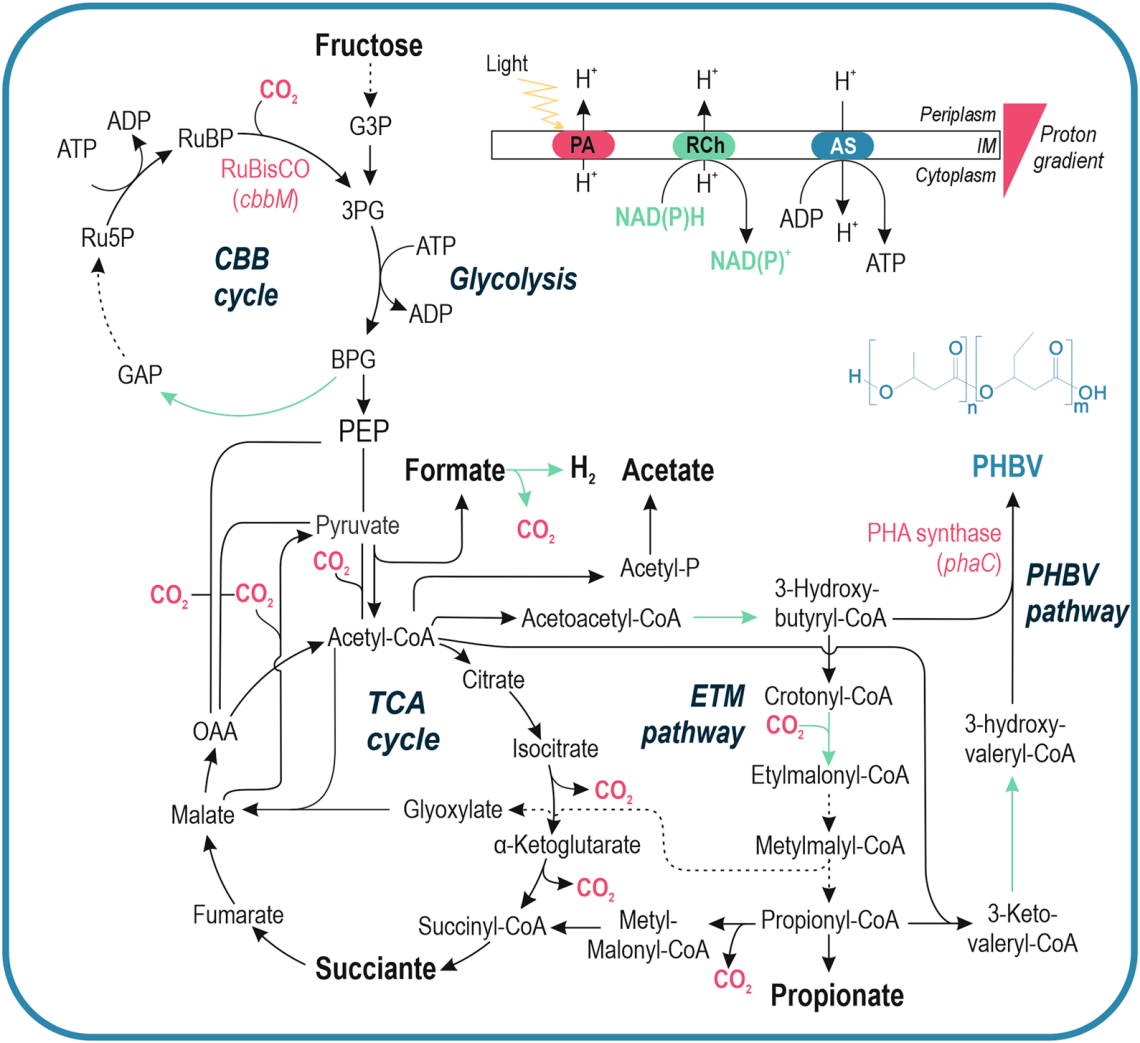
Scheme of the metabolism of *R. rubrum*. The main routes mentioned in this work are represented in the scheme: glycolysis, tricarboxylic acid (TCA) and Calvin–Benson–Bassham (CBB) cycles, Ethylmalonyl (ETM) and PHBV synthesis pathways. Energetic phenomena occurring in the periplasm and the inner membrane (IM), are depicted: cofactor recycling in the respiratory chain (RCh), the oxidative phosphorylation catalysed by the ATP-Synthase (AS) and the cyclic photosynthesis carried on by the photosynthetic apparatus (PA). The compounds measured in this work are highlighted in bold. Green arrows indicate NAD(P)^+^ regenerating reactions. G3P, glyceraldehyde-3-phosphate; 3PG, 3-phosphoglycerate; BPG, 1,3-bisphosphoglycerate; GAP, glyceraldehyde-3-phosphate; Ru5P, ribulose-5-phosphate; RuBP, ribulose-1,5-bisphosphate; RuBisCO, ribulose-1,5-bisphosphate carboxylase; PEP, phosphoenol-pyruvate

Species like the phototrophic purple non-sulfur bacterium (PNSB) *Rhodospirillum rubrum* can naturally produce PHBV due to the substrate specificity flexibility of its PHA synthase. *R. rubrum* is an alphaproteobacteria bacterium [12], with a very versatile metabolism (the main metabolic paths mentioned in this work are represented in Fig. 1). It is able to grow hetero- or autotrophically, using oxygen as final electron acceptor [13] or anaerobically, fermenting carbon sources [14] or extracting energy form inorganic sources, such as H_2_ [15] or carbon monoxide [16]. It also serves as a model organism for studies on nitrogen fixation [17–19], and pigments production [20, 21]. Some interesting aspects of its metabolism have been clearly explained in different articles [22–24].

When oxygen is present, aerobic growth in *R. rubrum* can be sustained by respiration in the absence of light. However, after oxygen becomes limiting, an incompletely known regulation system [25] remodels the energy and carbon processing machinery, to take metabolism network to a new optimal *statu quo*. Exposed to a source of light, cyclic photosynthesis can produce ATP. Molecular hydrogen breakage can provide electrons (reducing power) to eventually fix carbon dioxide by the well-known Calvin-Benson-Bassham cycle (CBB cycle). Fructose and other carbon sources (such as organic acids) can be consumed also in darkness. Even carbon monoxide, a highly toxic compound for many species, can be used as a carbon and energy supply [24]. As mentioned before, these metabolic scenarios start to be possible, and necessary for survival, when the oxygen level falls under a critical threshold, a condition *sine qua non* for avoiding lethal collateral effects from highly reactive chemical species called triplet bacteriochlorophyll and singlet oxygen [26]. Thus, *R. rubrum* can create extraordinary diverse metabolic contexts in its intracellular milieu that can be exploited to obtain industrially crucial bio-products, such as PHA [27].

The main player in the PHBV production strategies is by far *Cupriavidus necator*. This microorganism grows efficiently in minimal medium at 30 °C on a multitude of carbon sources [11]. The highest PHBV content with *C. necator* was obtained with a two-stage, fed-batch culture with glucose, propionic acid and limiting nitrogen conditions. The final co-polymer concentration was of 117 g·l^−1^, a polymer content of 74% CDW with a composition of a 14.3 mol% of 3HV [28]. With this general performance, *C. necator* stands out from other microorganisms like *R. rubrum*. However, *R. rubrum* seems to be a suitable catalyst for producing PHBV with high 3HV content [29]. It has been seen that *R. rubrum* cultures fed with 25 mM valerate (a C_5_ fatty acid) can produce up to 83% mol 3HV (23% of the cell dry weight). In this case, the requirement of bicarbonate as co-subtrate to stimulate the incorporation of C_5_ monomers was associated to the redox stress induced by the photoheterotrophic assimilation of valerate [29]. By using fructose, a carbon source with an even number of carbons (C6), Liu et al. reported a co-polymer production with 45% mol 3HV in 4-days bioreactor assays. This was the highest proportion of this monomer in the final PHBV produced by *R. rubrum* in the absence of C5 precursors [30]. However, no quantitative information regarding the polymer yield or total production was reported.

In this work, we delve into the potential that PNSB, and particularly *R. rubrum*, have on the production of PHBV from PHA unrelated carbon sources like fructose. We direct our study to microaerobic conditions and darkness. These conditions are particularly interesting from an industrial point of view since the aeration [31] and the exploitation of artificial light [32] result in investment and electricity expenditure which increase the final production cost. A reduction in the economic effort to obtain a co-polymer with optimal mechanical properties is thereby a major biotechnological challenge.

As initial step to fill this gap, we have explored the impact of aeration and light exposure in the PHBV accumulation of *R. rubrum* grown in shaken flasks. Different aeration levels were achieved by varying the agitation, the volume of the medium, and the gas insulation, to provide an exhaustive characterization. Light effect was also addressed given the relevance this factor has on *R. rubrum* physiology. We found that two-phase growth (aerobic-anaerobic) significantly improved the polymer accumulation at the expense of other components of the biomass. These results stand *R. rubrum* as a promising producer of high-3HV-content PHBV co-polymer from fructose.

## Results

### Exploring PHBV accumulation in different growth conditions

**Fig. 2:**
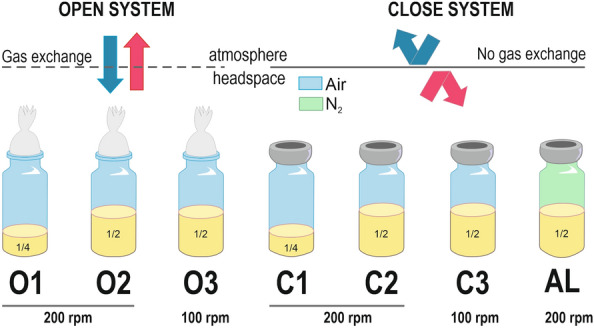
*Different conditions of the experimental set-up.* (A) Scheme of the experimental set-up. Open (O) and close (C) systems were established by using cotton or rubber plugs in order to control the exchange of gas with the atmosphere. The aeration levels were also controlled with the agitation (100 rpm or 200 rpm) and the filling volume (1/5 or 1/2). Medium in AL condition was degassed with N_2_ before autoclaving. All the condition, with the exception of AL, were grown in darkness

It has already been described the ability of *R. rubrum* to preferably accumulate PHBV, instead of the homopolymer PHB, when the appropriate substrate is supplied and the oxygen is limiting [^11^, ^23^, ^30^, ^31^, ^33^]. To explore the impact on PHBV composition and accumulation of growing *R. rubrum* in different aeration conditions, cultures were grown in the dark with modified RRNCO mineral medium containing fructose 13.3 mM and 1 g·l^−1^ of yeast extract. It is well established in the literature that the gas transfer from the gaseous phase to the medium in a shaken flask can be modified adjusting the experimental conditions [34, 35]. In this case, the aeration levels were controlled by means of (i) the agitation (100 rpm or 200 rpm), (ii) the filling volume (1/4 or 1/2) and (iii) the gas exchange of the system with the atmosphere (no exchange with rubber plugs, or free exchange with cotton plugs) (Fig. 2, Additional file 1: Table S1). In addition, an anaerobic culture (flushed with N_2_) exposed to white light (AL) was also included in the experimental design. The latter condition is anaerobic from the beginning of the culture and serves as a reference condition as it has been broadly studied [14, 36].

#### Cell growth and validation of the experimental set-up

**Fig. 3:**
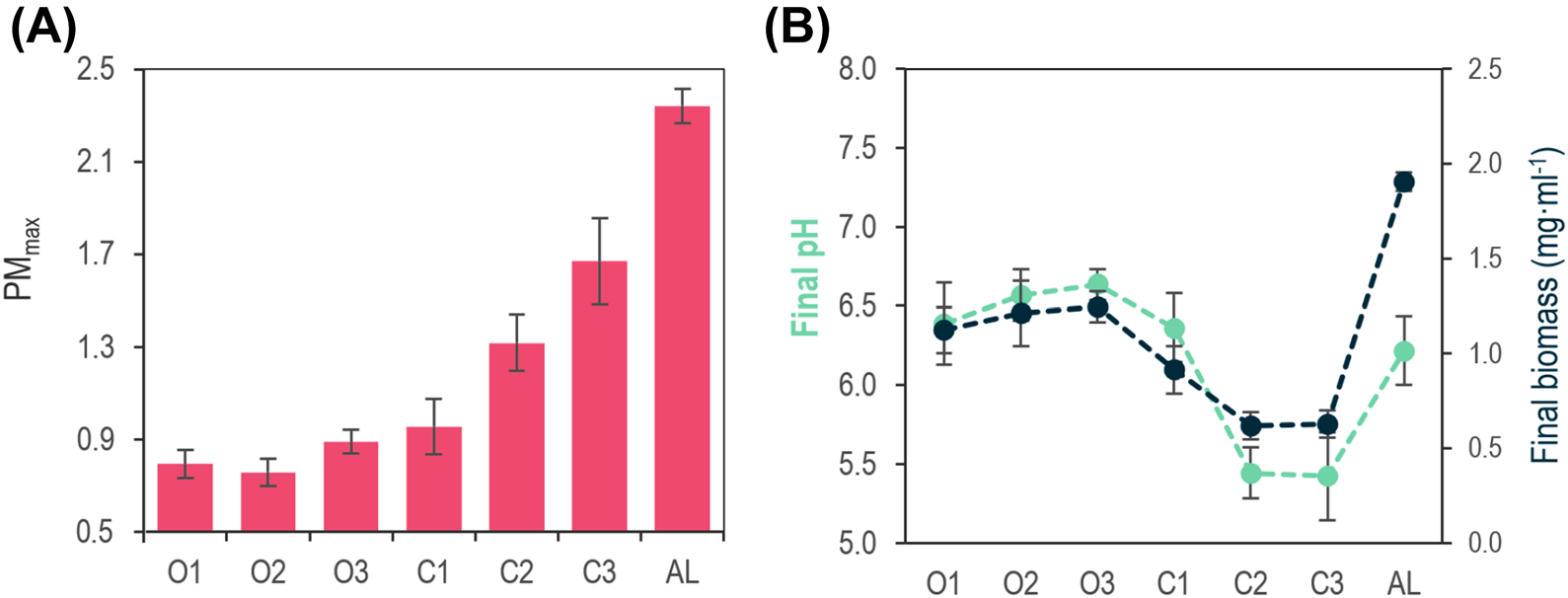
*Validation of the experimental set-up.* Seven different experimental conditions were tested. The factors manipulated to create a variety of conditions were the agitation (200 or 100 rpm), the filling volume (1/2 or 1/4), the gass interchange of the system with the environment (cotton or rubber plugs) and the presence or not of light. The experiment was carried out until the stationary phase of each condition. The PM_max_ production, final pH and biomass (mg·ml^−1^) were determined. Data is expressed as mean ± SD of triplicate determinations obtained from three independent experiments

The switch from an aerobic to a microaerobic/anaerobic condition triggers the photomembrane (PM) production in *R. rubrum*. This mechanism is thought to keep photosynthetic apparatus expression as low as possible when oxygen is present so as to minimize reactive species formation [23]. Thus, the photomembrane (PM) production is a critical parameter that manifests the aeration level sensed by the bacteria [37]. To test if our experimental set-up managed to create different aeration levels, the maximal production of PM was measured along the culture (Fig. 3A). PM production can be estimated by the ratio Abs_880nm_/Abs_660nm_. In parallel, the biomass at the stationary phase (6–10 days, depending on the condition Additional file 1: Fig. S1) and pH of the cultures were measured and monitored as a consequence of potential acidic metabolites secreted to the medium, typical of a fermentative metabolism (Fig. 3B).

The most aerated conditions (O1–O3) behaved similar in terms of maximal production of PM (PM_max_) (0.79–0.86), final biomass (1.12 mg·ml^−1^–1.25 mg·ml^−1^) and pH (from 6.4 to 6.7). Compared to them, the condition C1 resulted in a similar final pH (6.4 ± 0.2), slightly higher PM_max_ (1.05 ± 0.02) and lower biomass formation (0.91 ± 0.13 mg·ml^−1^). For the following less aerated conditions (C2, C3 and AL) PM_max_ is triggered, reaching the highest PM_max_ in the anaerobic condition with light (AL). The pH also drops concomitantly with the biomass in the conditions C2 and C3, being virtually the same between them (pH 5.4 ± 0.2 and 0.62 ± 0.07 mg·ml^−1^), but completely different compared to AL: the pH (6.2 ± 0.2) gets close to that observed in C1 and the biomass reaches 1.91 ± 0.05 mg·ml^−1^, the highest of all the conditions tested.

In general terms, the PM_max_, show a trend from more aerated to less aerated levels along the different conditions. However, considering the whole picture, some clear scenarios can be noticed: (i) aerobic growth where virtually no PM can be observed (O1, O2, O3), with a slightly acid pH (around 6.5) and intermediates levels of the total biomass formed at the end of the culture; (ii) microaerobic growth (i.e. cultures that initially had oxygen in the headspace but depleted it and developed high PM_max_ values) with acidification of the medium and the lowest final biomass production (C2 and C3); (iii) the anaerobic culture AL (the oxygen in the medium was depleted before inoculation) where the culture reached the highest PM_max_, final biomass formation and went slightly acid (pH > 6.0). We exclude condition C1 as it seems to be an intermediate condition between O3 and C2, with low but not neglectable PM_max_ values, and a pH level over 6.0.

#### 3HV monomers become the most abundant component of PHBV in low aeration conditions

To test the effect of the aeration level on the polymer production in our conditions, the end of the exponential phase was selected to measure the polymer content. This point was chosen since in preliminary experiments it showed to have the highest PHBV accumulation coincident with maximal turbidity (OD_660_ nm), which is related to the fact that the polymer content has an impact in the turbidimetric properties of the culture [38] (Additional file 1: Fig. S1).

**Fig. 4:**
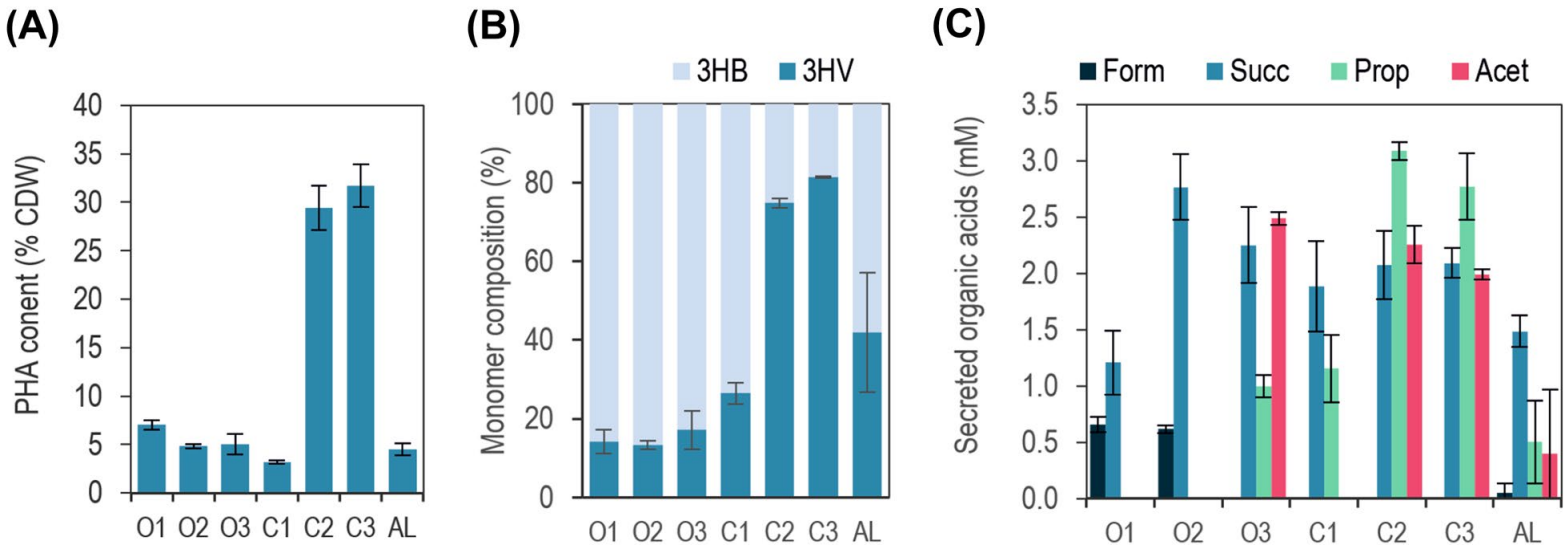
*PHBV accumulation and metabolites secreted.* The PHA content respect to the cell dry weight (CDW) (**A**), the proportion of 3HV monomer respect to the total polymer (**B**) and the metabolites detected in the culture supernatant (**C**), the samples were measured at the late exponential phase where the maximum polymer accumulation is observed. Cells were grown in RRNCO medium (fructose 13.3 mM) with an initial OD_660_ of 0.05. Seven culture conditions were tested: six were grown in darkness, being three of them open systems (O1, O2, O3) and three closed systems (C1, C2, C3). A forth closed system in the presence of light was included in the experimental design. Data is expressed as mean ± SD of triplicate determinations obtained from three independent experiments

Similar to the physiological variables studied in the previous section (Fig. 4, Additional file 1: Table S2), three different groups can be made on the bases of the amount and type of PHBV produced: (i) one group with high polymer accumulation (C2 and C3) with approximately 30% of PHBV and high 3HV proportion (up to 80%mol of 3HV in C3), (ii) a second group (O1, O2, O3 and C1) with low polymer accumulation (less than 10% CDW) manly composed of 3HB monomers (< 20%mol 3HV) and (iii) a third case (AL) also with low PHA content (4.5% CDW) but with almost equal proportion of each of them (43%mol of 3HV).

To have a cue on the metabolic routes undertaken in the different conditions by *R. rubrum* (Fig. 1), culture supernatant was analysed by HPLC as described in Materials & Methods. In agreement with previous reports, succinate, acetate, propionate and formate were found to be the major fermentative organic acids in the supernatant [39]. Succinate was present in all the conditions tested, being minimal in the most aerated level O1 (Fig. 4C, Additional file 1: Table S2). Low levels of formate were detected in O1, O2 and AL. On the contrary, propionate and acetate were most abundant in close systems grown in darkness (C2, C3) and in a lesser extent, in light (AL). Remarkably, propionate and acetate correlated to high 3HV content in the accumulated polymer. In this case, O3 and C1 resulted in intermediate situations. O3 had a secretion profile similar to that of close systems, and C1 secreted propionate but not acetate, opposed to C2 and C3.

### Transition from aerobiosis to microaerobiosis triggers PHBV accumulation over residual biomass

**Fig. 5:**
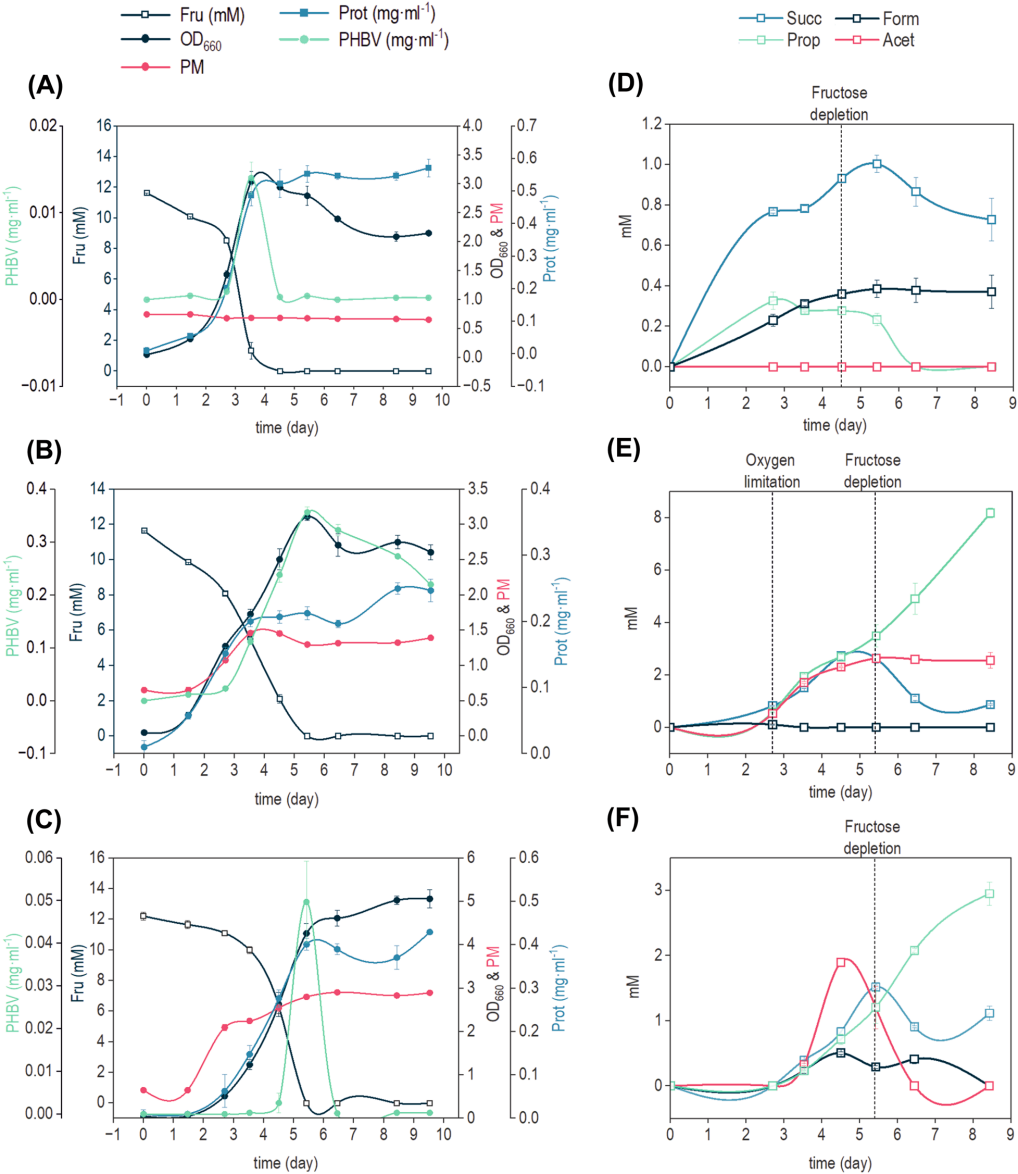
Growth kinetic and product formation in the selected conditions O2, C2 and AL. Cells grown in RRNCO medium with fructose 13.3 mM were inoculated at an initial OD_660_ of 0.05 and incubated at 30 ºC in constant agitation (200 rpm). Total protein (blue), turbidity (black full circles), PM production (red), fructose (dark blue, empty squares) and polymer amount (green), were measured along the experiment in O2 (**A**), C2 (**B**) and AL (**C**) conditions. Secreted succinate (light blue), propionate (green), acetate (red) and formate (dark blue) were quantified in the culture medium (**D**, **E**, **F** for O2, C2 and AL resp.). Representative curves of three independent experiments are expressed as mean ± SD

As the aim of this work was to find and study a set of experimental conditions to evaluate the aeration level impact in PHBV production, we decided to go deeper analysing conditions O2, C2 and AL as they presented well differentiated results in terms of polymer synthesis, but also in the other physiological parameters studied. We consider that condition O2 could be a good representative of the open systems and condition C2 was selected from the closed system conditions (it showed similar results to C3). AL was also kept in the experimental design since it offered a well-studied condition and a PM production as in the case of C2 (Fig. 5).

The evolution in PM production shows that in condition O2, *R. rubrum* did not perceive limiting oxygen levels along the experiment (Fig. 5). The only main change in the variables studied was given by the exhaustion of fructose, after which the succinate and the propionate, previously secreted, started to be consumed. Something similar occurred in AL. This condition showed 1 day of lag phase, after which the PM production started to increase together with biomass. Propionate and acetate were also synthetized. However, in this case, only acetate was consumed after fructose depletion. What occurs in C2 is slightly different given that during the first hours of the culture it can be considered an aerobic culture, as the headspace is composed mainly by air. At certain time-point between the first and second day, PM production was triggered, decreasing the growth rate and speeding up the production of acetate, propionate and succinate.

The most interesting behaviour, though, was observed for PHBV synthesis (Fig. 5, Additional file 1: Fig S2). For both O2 and AL, previously accumulated PHBV was rapidly mobilized after fructose was completely consumed. Curves of the polymer in these conditions describe a sharp peak just before fructose is over. In the case of C2, the heteropolymer PHBV was conspicuously accumulated after pigment synthesis was started, which reflects aerobic-microaerobic transition. Total protein is a good indicator of biomass [40, 41] specially when the optical properties, and thus the turbidity of the culture (measured with the OD_660_ in our case) may be affected by the presence of PHA [38]. After the mentioned transition, protein synthesis was dramatically slowed down, being the increase of turbidity in the culture manly attributable to PHBV stocking. Once the fructose was over, PHBV was gradually mobilized, describing a smooth ramp rather a peak as in O2 or AL.

**Fig. 6:**
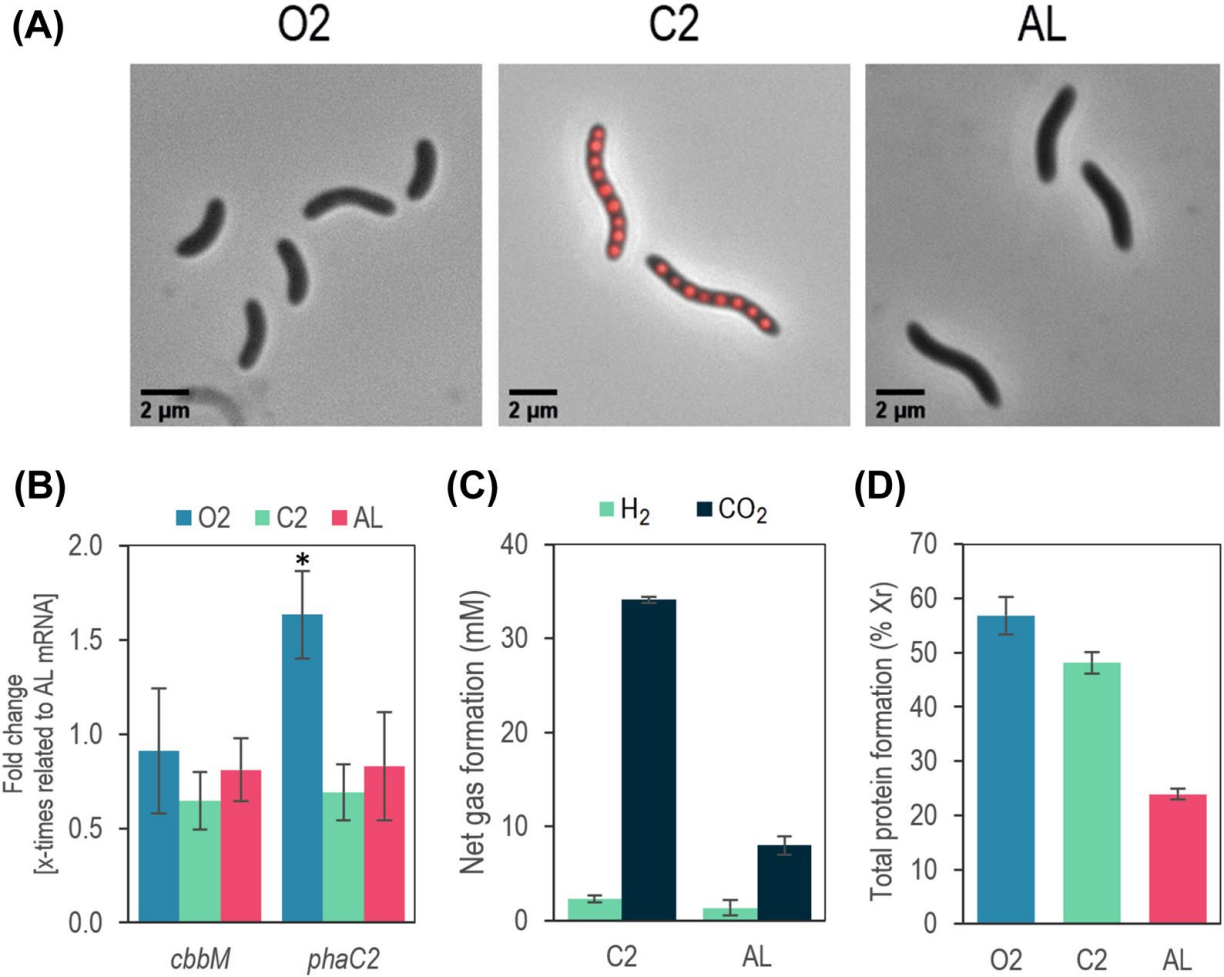
*Physiological characterization of R. rubrum in conditions O2, C2 and AL.* **A** Nile-red stained cells under the optical/fluorescent microscope. Only condition C2 lead to observable PHBV granules. Pictures were taken at the moment of maximal PHBV accumulation. **B** RT-qPCR of *phaC2* (PHA synthase) and *cbbM* (RuBisCO) in the exponential phase of growth (in the case of C2-grown cells, after they have started PM production (3 days). The transcriptional levels of *phaC1* and *phaC3* were undetectable (C_T_ > 38). Fold change respect to the condition AL is represented. Statistical analysis was performed using one-way ANOVA followed by Dunnett’s comparison test. A significant effect was indicated by * (P < 0.05). **C** Net gas production at the stationary phase (10 days) of C2- and AL-grown cultures. The concentration of the gas was calculated as the total amount (mmol) of gas produced divided by the culture volume, to make it comparable to the other metabolites measured and the carbon source. **D** Total protein content measured by Bradford method. The percentage is referred to the residual biomass (Xr), i.e. without PHBV

Differences in polymer content was clearly reflected in the presence of conspicuous granules aligned along the major axis of the cell (Fig. 6A). In this condition the cells reached 4.9 ± 1.2 µm in length, similar to AL-grown cells (4.4 ± 0.9 µm) and almost 69% longer than cells cultured in condition O2 (Additional file 1: Fig. S4), although PHBV was not observable in the latter. The total protein content of the residual biomass (i.e. the total biomass minus PHA) was also different for the different conditions (Fig. 6D). A higher protein content was observed in aerobically grown cells (57 ± 3%) slightly higher than C2-grown cells (48 ± 2%, p < 0.02). In the case of the photoheterotrophic culture, the protein content was much less than the previous (24 ± 2%). While, the whole cell spectrums of C2- and AL-grown cells indicate a similar composition of the PM [20] (Additional file 1: Fig. S4), the PM level of the latter is much higher at the expense of other biomass constituents.

Three genes encoding for this function (*phaC1*, *phaC2* and *phaC3*) were identified in *R. rubrum,* being gene *phaC1* (Rru_0275) the only one located in a putative PHB operon. Paradoxically, even though PhaC2 had a significantly lower enzyme activity than the other two synthases, it was detected intracellularly in much higher concentration than its homologous. Moreover, multiple and single deletion of the genes *phaC1*, *phaC2* and *phaC3* showed that PhaC2 was the main synthase involved in PHB synthesis with acetate [27]. The polymers accumulated in these conditions were manly composed of 3HB, although small quantities of 3HV and 3HHx (C_6_) (< 6%) were observed with hexanoate as single carbon source [27]. To ascertain which synthase was principally responsible for the different conditions, mRNAs from genes *phaC1, phaC2* and *phaC3* were tested with RT-qPCR (Fig. 6B). In accordance to Jin’s experiments, only *phaC2* transcript was detectable in the RNA extracted in our conditions, being those of *phaC1* and *phaC3* undetectable (C_T_ > 38). Remarkably, O2-grown cells presented higher relative values of this transcript than C2- and similar to AL-grown cells. This result, together with the secretion profiles of acetate and propionate, show that polymer composition is determined mainly by substrate availability and not by the action of PHA synthases with different substrate affinities.

### After PHBV accumulation CBB cycle, more than hydrogen production, is needed for redox cofactor poise

PNSB count on several “electron sinking” strategies to mitigate the excess of electrons, such as CO_2_ and N_2_ fixation, and the production of H_2_ and PHA [22]. In the case of C2-grown cells, PHBV accumulation levels suggest that its synthesis may serve as an important electron sink after oxygen depletion (Fig. 5C). The little content of hydrogen in the head space of the closed systems C2 and AL (< 2 mM) (Fig. 6C) at the end of the culture indicates that H_2_ evolution might not have been the main strategy for redox cofactor recycling in these conditions. While nitrogen fixation is strongly repressed under fermentative conditions in darkness when NH_4_^+^ is present [19, 42], CBB could play a role in preserving the redox poise in our selected conditions. To test this hypothesis, the transcription level of its key enzyme, the ribulose-1,5-bisphosphate carboxylase-oxygenase (RuBisCO), encoded in *cbbM*, was measured by RT-qPCR (Fig. 6B). In our three growth conditions, we observed appreciable levels of *cbbM*. Narancic *et al.* has already described that CBB cycle can be active even during aerobic conditions in *R. rubrum* [13]. In our results, its relative expression level in O2-grown cultures was comparable to that measured in conditions C2 and AL. This indicates that once the cells have adapted to microaerobic conditions (C2), CBB may play a role in cofactor re-oxidation together with PHBV synthesis.

### CO_2_ accelerates the adaptation to low oxygen tensions in the absence of light but only promotes PHBV accumulation over residual biomass

From an industrial point of view, tailoring 3HV proportion becomes very relevant since the final polymer composition defines its mechanical properties and potential applications. It is described in the literature that the rate of propionate and succinate production is dependent on the CO_2_ concentration of the medium [43]. As the secreted propionate and valeryl-CoA, the C_5_ constituent of PHBV, both share the same precursor, propionyl-CoA, we speculated that the availability of the latter could be influenced in a dose dependent manner by the concentration of CO_2_, affecting the 3HV %mol of the final monomer. To test this hypothesis, we washed and transferred O2-grown cells in the exponential phase (OD_660_ = 1.0–1.5) to fresh anaerobic medium containing 40 mM fructose and different bicarbonate concentrations (0.0 mM, 3.0 mM, 6.0 mM and 12.0 mM) in darkness. Thus, we could eliminate CO_2_, and emulate the aerobic-anoxigenic transition that C2-grown cells undergo but controlling the CO_2_ present in the medium.

**Fig. 7:**
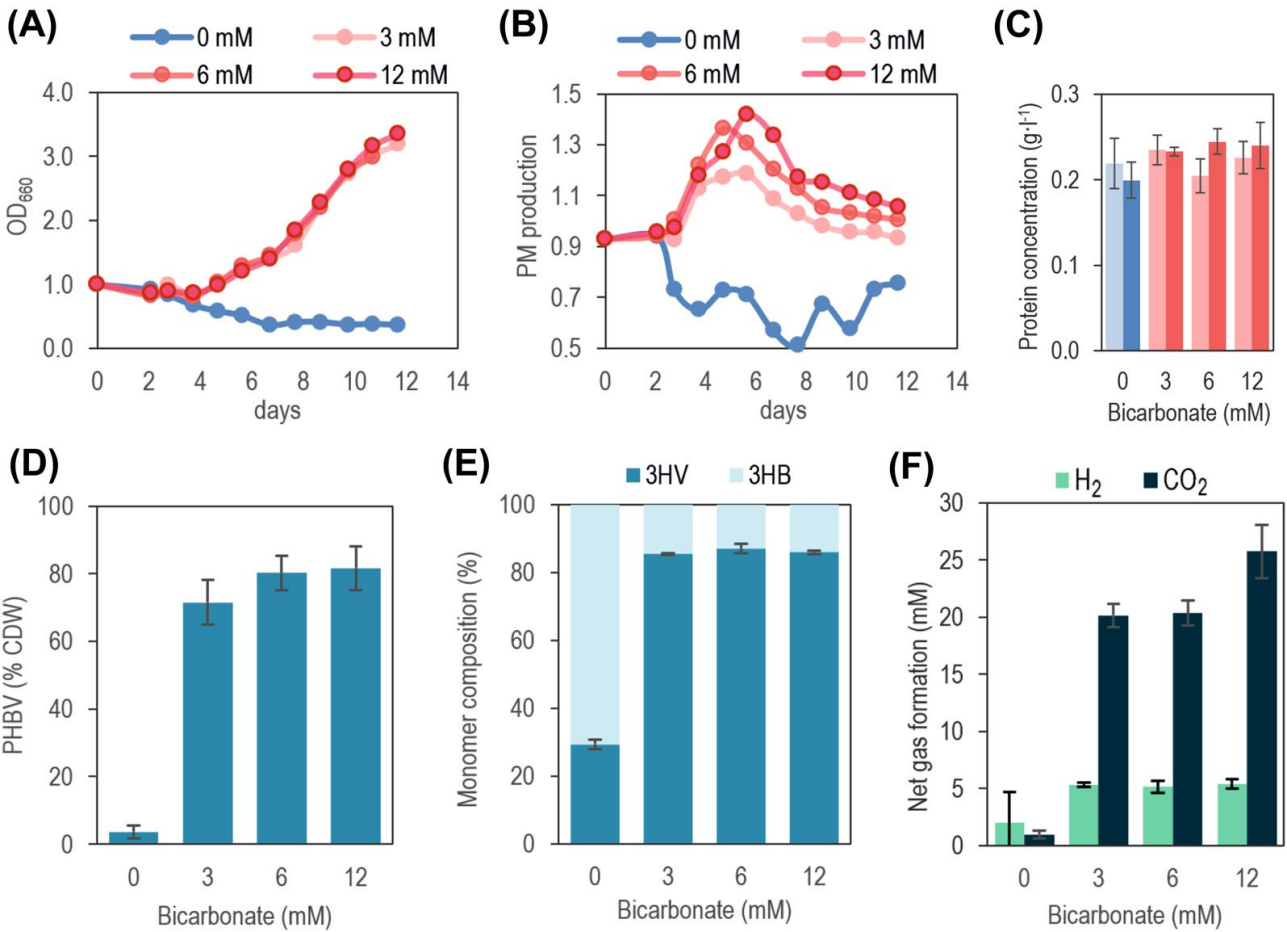
Transition from aerated to anaerobic cultures. O2-grown cells were transferred to anaerobic bottles with fresh RRNCO medium, and 40 mM fructose. Bicarbonate was added (0.0 mM, 3.0 mM, 6.0 mM, 12.0 mM) and the pH adjusted to 7.0 with HCl 0.1 N. **A** Growth curves of OD_660_ and **B** PM production. Only the cultures with bicarbonate were able to grow in the lapse of the experiment. PM production depended upon bicarbonate concentration. **C** Total protein measured with the Bradford method of cells in time-point cero (light blue or light red) and at the end of the experiment (dark blue or red). **D** PHBV accumulation and **E** 3HV %mol was the same for all the conditions as long as they had bicarbonate. **F** Production of CO_2_ and H_2_ was quantified, resulting in a net accumulation of both gases in the headspace of the bottles. The concentration of the gas was calculated as the total amount (mmol) of gas produced divided by the culture volume, to make it comparable to the other metabolites measured and the carbon source

Interestingly, no growth was observed in the absence of bicarbonate after 12 days nor PM production was observed (Fig. 7A). This indicates that CO_2_ is required immediately after oxygen depletion to go on with the physiological adaptation to anaerobic conditions. In the presence of bicarbonate, PM production was triggered after the third day of culture. Moreover, the Abs_880nm_/Abs_660nm_ ratio was higher with higher concentrations of this compound from the fifth day. As expected, total protein was invariable in all the conditions (Fig. 7C). Contrary to our hypothesis, 3HV %mol did not respond proportionally to bicarbonate concentration or, what is equivalent, to the initial amount of CO_2_ (Fig. 7D). Surprisingly, PHBV content reached up to 81% CDW with 12.0 mM of bicarbonate, and the highest 3HV %mol of all the studied conditions (86%mol) (Fig. 7E). Cells also produced hydrogen and CO_2_, even without bicarbonate, and consumed up to 2.5 ± 0.1 mM of fructose, showing they were metabolically active at least in a basal level (Fig. 7F, Additional file 1: Table S3). The carbon balance considering all the metabolites measured (organic acids, PHBV, and net CO_2_), had a good fitting (around 6–14% around the 100%) (Additional file 1: Table S3). The imperative requisite of CO_2_ for adaptation from aerobic to anaerobic conditions further confirms the presence of an active CBB cycle at high oxygen levels.

## Discussion

In the present work, different metabolic scenarios were explored with the principal aim of studying PHA accumulation in *R. rubrum*. Light was also included as photoheterotrophic growth is well characterized in PNBS [16, 44–46]. This is how a particularly interesting aeration condition, from the polymer accumulation perspective, arose: the microaerobic condition C2 (and C3). C2 represents the transition from an aerobic metabolism (such as in O2) to a potentially heteroautotrophic one, even though light is not present, with intermediate characteristics of both. After oxygen had fell under a threshold, the genetic regulatory system triggered the synthesis of the photosystem apparatus, reflected by the increase of PM. In nature, this mechanism could be a smart pirouette to overcome bacteria need of surviving in the absence of electron acceptors, waiting for a source of light to come out with a complete stock of carbon reservoir ensured in their cytoplasm. The issue of redox poise, residual biomass vs polyester formation, and the biotechnological relevance of these findings, are discussed in the following lines.

### Calvin–Benson–Bassham cycle as the main pivot for metabolic tacks

The biochemical constraints of a metabolic network are generally determinant of the overall need for electron carrier oxidation [47]. This fact becomes especially critical when cells are challenged with a sudden change in the environmental conditions. Dissipating excessive reduced electron carriers is crucial for growth when respiration is not permitted. This re-establishes the pool of oxidized cofactors to go ahead with biomass formation. We showed that *cbbM* (Rru_A2400) coding for ribulose 1,5-bisphosphate carboxylase (RuBisCO) large subunit, was actively transcribed in all the studied conditions, including aerobic growth. The presence of RuBisCO activity in aerobic conditions was described for the first time some years ago. While Grammel found rubisco activity only in phototrophic cultures [48], Narancic showed that RuBisCO, was active in *R. rubrum* under aerobic growth in the dark [13].

This fact explains why O2-grown cells were so dependent on bicarbonate to get adapted to anaerobic conditions. The so-called closed systems (C1, C2, and C3) differ from the open systems (O1, O2 and O3) in the gas exchange with the environment. While O_2_ is constantly being replenished and CO_2_ dissipated in the later (offering a relatively stable condition), in the closed systems their partial pressures evolve inversely throw the experiment. This fact leads to the depletion of O_2_ and the accumulation of CO_2_, affecting negatively cell growth by decreasing the pH of the medium. Paradoxically, CO_2_ becomes vital for getting adapted to the absence of oxygen. Even though cultures form aerobic and microaerobic condition (e.g. O2 and C2), behaved similarly during the first hours, after the oxygen tension fell below a critical level, C2-grown cells made a sudden change in their metabolic choice. Residual biomass formation was virtually supressed and the co-polymer PHBV was accumulated. These facts were accounted by the total protein and the PHBV curves (Fig. 5).

The importance of the Calvin–Benson–Bassham (CBB) cycle for resetting the intracellular redox poise has been profusely reported. This is particularly true for PNSB bacteria growing in photoheterotrophic conditions. In this case, CO_2_ serves as the electron sink [47, 49, 50]. When we transferred O2-grown cells directly to anaerobic conditions in the dark (similar to what can be expected in a late C2 condition), they synthesised pigments but most of the carbon assimilated was dedicated to PHBV, tied to the availability of CO_2_ (bicarbonate) in the medium. In the case of CO_2_ scarcity, they remained like resting cells with a minimal metabolism, suggested by the detection of small quantities of H_2_ and CO_2_ in the headspace after 13 days of culture. It could be predicted that after a critical amount of this gas was accumulated, they could have started with the adaption to the anaerobic conditions and eventually accumulate PHBV.

It must be mentioned that the role of CBB cycle in photoheterotrophic conditions has been somehow controversial. A kinetic model of the CBB cycle based on the central carbon metabolism of the cyanobacterium *Synechocystis* sp. PCC 6803 showed that the accumulation of some intermediates, such as those of ribulose 1,5-bisphosphate (RuBP) over saturation levels may lead to instability problems. Thus, modifications in the metabolism that increase the amount of RuBP, or repression of Rubisco, would stun the metabolic network [51]. From this point of view, CO_2_ scarcity would impose a hurdle, not because of an imbalance in the redox state, but by preventing the carboxylation of RuBP and its resulting accumulation, with detrimental effects on the cell physiology. This toxic effect of RuBP was also described for *R. rubrum* [52], where toxicity resulting from an elevated intracellular pool of RuBP was associated with an impaired balance between phosphoribulokinase (PRK) and RuBisCO. Qualifying these results, McKinlay demonstrated that *prk* mutants of *R. rubrum* and *Rhodopseudomonas palustris* (unable to produce RuBP) cannot grow photoheterotrophically on succinate unless an electron acceptor is provided or H_2_ production is permitted [53]. Thus, the Calvin cycle is still needed to oxidize electron carriers even in the absence of toxic RuBP. In conclusion, a probable solution for this controversy is a combination of both explanations: CBB cycle could contribute to adaptation and biomass formation by re-establishing redox balance and by preventing RuBP accumulation. In our experiments, the need of having an active CBB cycle during aerobic-anaerobic transition was demonstrated by the mentioned CO_2_ dependency of this adaptation process.

### PHBV synthesis pathway is an essential route for dissipating redox potential when fructose is the carbon source in dark anaerobic conditions

PHA [54, 55] and hydrogen [56] metabolism plays a key role as a regulatory mechanism for the optimization of carbon and energy flow in the cell. When fructose was used as the carbon source in our experiments, the main fermentative routes in low oxygen tensions tend to produce more acetate and propionate, and almost no formate. A similar behaviour was described by Grammel [48]. He observed an active TCA cycle even in their microaerobic conditions (0.5% pO2), were the reductive branch was predominant and resulted in propionate accumulation and absence of formate. The latter can be oxidized to release H_2_ by the pyruvate-formate lyase, as described for cells transferred from aerobic to anaerobic conditions in the dark using pyruvate as carbon source [57]. However, as only little amounts of formate were detected in our system (which has fructose as carbon source), there is little precursor available for H_2_ production, and thus, the metabolic framework needs to dispense the excess of reduced cofactor by further reducing propionate and acetate into PHBV.

In this sense, McKinlay and co-workers identified two metabolic factors that help explain variable H_2_ yields among different PNSB: (i) the route taken to generate biosynthetic precursors and (ii) the amount of competing Calvin cycle flux [56]. In microaerobic conditions (such as C2), we could see that one of the main components formed after oxygen depletion was PHBV, indicating that PHBV was a crucial route for dissipating redox potential. The composition of the polymer formed can also be seen as a result of the maximization of re-oxidation of electron carriers. If we consider the basic formula of both monomers constitutive of PHBV co-polymer, 3HV has an oxidation state of −0.8 while that value for 3HB is −0.5. Then, a higher amount of oxidized electron donors could be regenerated by increasing the proportion of 3HV in the polymer. Following McKinlay’s reasoning, after oxygen depletion, the initially aerobic culture found in PHBV-synthesis path a convenient and efficient shortcut to maintain an active metabolism, pending on the conditions to change and turn residual biomass formation once again possible.

Finally, it is worth noting that the only polymerase actively transcribed in our three selected conditions was exclusively PhaC2, which has already been described in heterotrophic conditions [27], indicating that the polymer composition in the conditions studied is not the result of different enzymes having particular substrate affinities but an effect of having the same enzyme ready for on-demand polymerization of the available monomers provided by the central metabolism.

### ***R. rubrum*** as a powerful biocatalyst for high C_5_ content PHBV

PHAs, whose properties depend on the composition, can perfectly compete with many of the conventional petrochemically-based plastics [58]. P3HB, the naturally most abundant type of PHA, is synthesized by numerous wild type and recombinant organisms at high yields. However, practical applications of the P3HB homopolymer are limited mostly due to its high crystallinity and concomitant stiff and brittle characteristics [59].The tensile and impact strength as well as the flexibility, can be increased incorporating 3HV to the polymer so as to decrease the crystallization of the 3HB grid [60]. With a composition of 3HV of 24 mol%, PHBV was first commercialized by ICI (Zeneca) in 1970 under the trade name Biopol [59].

In our best condition (i.e. O2-grown cells transferred to anaerobic conditions in the dark with 40 mM fructose), a PHBV co-polymer composed of 86% 3HV mol% was produced up to 81% of the total cell dry weight (CDW), resulting in a volumetric production of 0.78 ± 0.07 g·l^−1^. More interestingly, we obtained high PHBV yields with fructose, a non C_5_ skeletons carbon source. As mentioned in the introduction, valerate is the conventional carbon source when looking for PHBV accumulation by *R. rubrum*, reaching up to 83%mol 3HV. The specific production was, though, quite low (23% CDW). Liu *et al.* used fructose to produce 45% mol 3HV in 4-days bioreactor assays [30]. The highest 3HV %mol found in the literature corresponds to cultures of Serratia sp [61] and *Aeromonas hydrophila* [62] with 100% %mol (homopolymer) in both, obtaining 0.33 g·l^−1^ and 3 g·l^−1^ after 10 days and 1.7 days respectively. When *A. hydrophila* was cultured in a 6L bioreactor, the same authors obtained 60% CDW (23 g·l^−1^) of PHV. The carbon sources used were glucose and undecanoic acid in a two-step fermentation scheme.

We also found that *R. rubrum* can virtually behave as resting cells in a complete medium after oxygen is depleted, with minimal energy and carbon requirements for physiological adaptation (to anaerobiosis) and cell maintenance. This curious feature could be exploited in two-phase bioreactor assays to optimize and scale the production of PHBV independent of light, with the only requirement of supplying CO_2_, fructose, and probably minimal quantities of phosphate and nitrogen. It is still needed to explore inexpensive fructose-containing feedstocks such as industrial molasses, which can contain up to 20% of its carbon in form of fructose [14].

## Conclusion

By exploring different metabolic scenarios of the versatile species *R. rubrum*, we found that the redox poise constraints in anaerobic cultures in the dark minimize residual biomass and optimize PHBV accumulation with high 3HV %mol content. The adaption from aerobic growth to anaerobic conditions (either because of oxygen full consumption by the cells or by direct transfer from one condition to the other), has the *sine qua non* requirement of CO_2_ at critical amounts to boost the crucial CBB cycle, which is active even in the aerobic phase of growth. These findings open new opportunities to the synthesis of tailored-made co-polymers of PHBV in cost-reduced fermentation set-ups.

### Materials and methods

#### Growth conditions

The strain used in this work was *R. rubrum* S1 (ATCC 11,170, DSMZ 467 T). It was preserved in Luria–Bertani (LB) medium [63] with glycerol (15% v/v) at −80 °C and recovered in LB fructose (10 mM). For the experiments shown in Results, RRNCO medium [16] was used with minor changes (1 g·l^−1^, K_2_HPO_4_ 19.1 mM, pH 7.0). The precultures were cultivated in LB (25 ml) using closed 50 ml-falcons and incubated 24 h at 30 °C and 200 rpm in darkness. The starting OD_660_ was 0.05. The experimental cultures were done in penicillin bottles using cotton plugs or sealed with 20 mm-thick chlorobutyl plugs (Wheaton^®^ W224100-202). They contained 1/2 or 1/4 of its volume occupied with medium and were incubated at 30 °C with 100 rpm or 200 rpm of agitation depending on the experimental condition. For anaerobic cultures, bottles half-filled with medium were heated (70–80 °C) using a microwave oven machine and degassed during 15–20 min with N_2_. Phosphate (19,1 mM), fructose (13,3 mM) and the reducing agent sodium sulfate (0,01%) were added with a syringe before the inoculum. Photoheterotrophic cultures were performed at 30 ºC with 200 rpm of orbital agitation and the illumination intensity was 1,5 klux. A scheme of the complete experimental set-up is depicted in Fig. 2 and Additional file 1: Table S1.

For aerobic-anaerobic transition experiments, cells grown aerobically (condition O2) in modified RRNCO medium (fructose 13.3 mM) were harvested after reaching an OD_660_ 1.0. They were washed with saline solution (NaCl 0.85%), and concentrated to an OD_660_ of 10 to be transferred anaerobic modified RRNCO medium (fructose 40 mM) with a syringe (initial OD_660_ of 1). The bicarbonate, previously degassed with N_2_ and filtered to sterility (Syringe Filter 0.22 µm, Minisart^®^) was added to reach a final concentration from 3.0 mM to 12 mM. The pH was neutralized with HCl 0.1 M.

#### Quantification of polyhydroxyalkanoates

The cellular PHBV content and proportion of the monomers that constituted the polymer were determined by methanolysis. Briefly, this procedure was carried out by suspending 2–5 mg of lyophilized cultured pellets into 2 ml of methanol containing 15% of sulfuric acid and 2 ml of chloroform containing 0,5 mg/ml of 3-methylbenzoic acid (3 MB) used as an internal standard. The mixture deposited at glass screw-cap tubes (Boro 3.3, Pirex^®^) was then incubated at 100 ºC for 4 h using thermoblocks (QBD4 Digital block heater, Grant). After cooling on ice, 1 ml of distilled water was added in order to separate cellular debris and the sulfuric acid from the organic phase with the methylated monomers. After a centrifugation (1 min, 3500 rpm), that facilitated the separation of the phases, the aqueous phase was removed. This process was repeated twice to ensure complete removal of the aqueous residues from the mixture. Finally, a small amount of Na_2_SO_4_ powder was added to dry the chloroform phase to prevent water entering the chromatography column.

For the analysis of methanolized samples an Agilent (Waldbronn, Germany) series 7890A gas chromatograph coupled with a 5975C MS detector (EI; 70 eV) and a split–spitless injector were used. An aliquot (1 ml) of organic phase was injected into the gas chromatograph at a split ratio of 1:50. The column used was DB-5HTDB-5HT (400 °C; 30 m by 0.25 mm by 0.1 µm film thickness). The mobile phase was helium gas and was injected at a flow rate of 0.9 ml·min^−1^. The injector and transfer line temperature were set at 275 °C and 300 °C respectively. For efficient peak separation, the oven temperature program was set to start at 80 °C for 2 min and then rise to 175 °C at a rate of 5 °C·min^−1^. Mass spectra were recorded in full scan mode (*m/z* 40 to 550). Samples with known concentrations of commercial PHBV polymer (Sigma Aldrich^®^) were used to calculate the process efficiency. The retention time for each methylated monomer obtained in this work was 2,6 min (C_4_), 3,8 min (C_5_), and 7,7 min (3 MB, internal standard).

To test the evolution of PHBV content in the selected conditions (O2, C2 and AL), the polyester content was quantified by the hydrolyzation and transformation of the constitutive monomers, 3-hydroxybutyrate (3HB) and 3-hydroxyvalerate (3HV) into crotonic acid and 2-pentenoic acid, respectably [64]. This technique was used since it requires smaller sample volumes and gives accurate results. 400 μL of culture were centrifuged (2.700 rcf, 5 min), and the pellet resuspended with one volume of NaOH (1 M). The resuspended cells were heated in a thermostatic block (94 °C, 1 h) and neutralized by adding the same volume of HCl (1N). Adipic acid (10 mg ml^−1^) was added to HCl as an internal standard. The neutralized samples were centrifuged (2.600 rcf, 10 min) and the supernatants were passed through 0.45 μm pore filters (13 mm, Avantor Ref. No. 514-0069). The crotonic acid and 2-pentotenic acid were determined by HPLC as explained below. A treated standard curve of commercial PHBV (Sigma Aldrich^®^) dissolved in chloroform (0.5 g·l^−1^) was used to interpolate the concentration of the experimental samples. Samples corresponding to the maximal accumulation point of PHBV were analysed in parallel by mathanolysis in order to validate the results.

#### High performance liquid chromatography determinations

Culture samples containing the compounds of interest (fructose, succinate, formate, acetate and propionate), were centrifuged and the supernatant was filtered as in the case of PHA hydrolysates (see above). They were analysed by HPLC (1260 Infinity II LC System, Agilent^®^) with an Aminex HPX-87H column (Biorad, Hercules, CA, USA) heated to 50 °C. The mobile phase was H_2_SO_4_ (5 mM) and the flow rate used was of 0.6 ml·min^−1^. The detection was performed by a refractive index detector and UV at 210 nm. The compounds were identified by their retention time, and the concentration was estimated with standard curves prepared with HPLC-grade compounds. In the case of PHA hydrolysate samples containing crotonic and 2-pentenoic acids, the same chromatographic conditions were used but the column was heated to 60 °C. In this case, the peaks were detected with the UV-detector.

#### Gas analysis

The analytical protocol for H_2_ and CO_2_ quantification has been described elsewhere [15]. Ne (p_Ne_ = 0.33 bar) was used as the internal gas standard. Calibration curves of H_2_ and CO_2_ were performed by representing the ratio of the partial pressures of the compounds and the internal standard (pi /pNe) with respect to the ratio of areas provided by the chromatogram (Ai /ANe). For the sake of clarity, the amount of gas is represented as if the total amount of each gas in the headspace were content in the volume of the medium, leading to mM units.

#### Spectrophotometric techniques

Cell growth was followed by the turbidity of the cultures, the measure of its optical density at 660 nm (OD_660_) was made in 96-well plates with a MultiskanSky spectrophotometer (Thermo Fisher Scientific^®^). The complex antenna levels, also called here as photo membrane (PM), were estimated by normalizing the absorption at 880 nm by the OD_660_ (Abs _880 nm_/Abs_660_) [36] with the same equipment. The absorption spectra of intact cells were determined by using 2 mm path-length cuvettes with a Shimadzu UV-1900i spectrophotometer. Pelleted cells were re-suspended in a volume of glycerol 80% so as to obtain a final OD_660_ = 1 [20].

#### Biomass and total protein determination

Biomass was measured gravimetrically by weighing the lyophilized pellet of the cultures. Total protein content was determined by Bradford method [65] using 20 µl of sample in 200 µl of reactant in a 96-well plate and measured with a MultiskanSky spectrophotometer. The standard curve was done with known solutions of bovine serum albumin (Merk, A9418).

#### Real time PCR assay

Total RNA was extracted from *R. rubrum* cultures grown under the experimental conditions described before (O2, C2 and AL) and harvested at the exponential phase (OD_660_ 1.5–2.0). The medium used in all cases was RRNCO with fructose (13.3 mM) as carbon source. For the extraction, the procedure described in High Pure RNA Isolation Kit, (Roche^®^) was followed, after which an additional DNA digestion was carried out by adding DNAse (Ambion^™^) and incubating the mix at 37 ºC for 30 min. The absence of DNA in the RNA samples was verified by PCR. Finally, the concentration and purity of the RNA samples were measured by using a ND1000 spectrophotometer (Nanodrop Technologies) and its integrity was checked by electrophoresis in agarose gel 0,7%. For cDNA synthesis, 1 µg of RNA was taken as template and the kit (Transcriptor First Strand cDNA Synthesis Kit, Roche^®^) was used. Random hexamer primers were used to perform the reversed transcription reaction. The mix was incubated at 55 °C for 30 min followed by 5 min at 85 °C to inactivate the reverse transcriptase. cDNA samples concentration was measured using a ND100 Spectrophotometer (Nanodrop Technologies) and a PCR was performed using the primers designed for de RT-PCR to check amplification.

Real-time PCR was carried out using SYBR Green technology, the measuring instrument was the LightCycler 480 II (Roche^®)^. The synthesized cDNA was used as a template. The amplification program used was as specified for the SYBR Green mix: first a preincubation at 95 ºC for 5 min, followed by 40 cycles 10 s at 95 ºC, 20 s at 56 ºC and 30 s at 72 ºC; in the last step a melting curve was made by heating the sample up to 97 ºC. For the measurement of efficiency of the primers standard curves were made with dilutions of *R. rubrum* genomic DNA. Target primers were (5´→3ʹ): O279 (CCCCTTGGCCAATATCCGAC) and O280 (TCGTTGAGCCAGTCTTCGAC) for *phaC1* (*Rru_A0275*); O281 (AGTCTTGCCACAGGCTCATC) and O282 (CAATCAGGGCAGCGAGAAG) for *phaC2* (*Rru_A2413*); O283 (TTTATGGGCGTCAGGTGGTC) and O284 (AACCTCGAAGGCGGTTTCAT) for *phaC3* (*Rru_A1816*); O295 (ATATGGCTATGTGGCGACCG) and O296 (CCGGATAGGCGATCTTGGTC) for *cbbM* (*Rru_A2400*). 16 s rRNA gene (Rru_AR0004) was used as the reference gen, using the primers O293 (AAGAAGCCCCGGCTAACTTC) and O294 (CTGGGAATTCCACCACCCTC). A relative quantification was done following the 2-ΔΔCT method [66]. This analysis was performed in three technical replicates from three independent biological samples.

#### Image processing

10 µl samples of cultures grown in conditions O2, C2 and AL were harvested at the late exponential phase (time of maximum PHBV accumulation) and deposited on microscope slides (Marenfeld). The samples were fixed with heat (passing gently once or twice the slide over the flame) and stained with a solution of Nile Red (0.5 μg·ml^−1^). The stained cells were observed under an optical microscope (DM4 B, Leica Microsystems). For fluorescence imaging a laser was used (pE-300^lite^, CoolLED). Photographs were taken with a camera attached to the microscope (DFC345 FX, Leica Microsystems). The processing of the images was carried out with ImageJ software.

### Supplementary Information

**Additional file 1: Table S1.** *Summary of the conditions for the open and closed systems.* **Table S2.** *Biomass in the late stationary phase and PHBV production and metabolites secreted to the supernatant in the early stationary phase.* **Table S3.** *Metabolite production and substrate consumption.* **Figure S1.** *Growth curves of R. rubrum in different aeration levels.* Open systems O1 (A), O2 (B), O3 (C), and closed systems C1 (D), C2 (E), C3 (F), were grown in darkness, and the anaerobic culture AL (G) was grown with white light. The turbidity (blue curves) is depicted in the same scale as PM production (red curves). Points in the late exponential phase and early stationary phase were taken to assess PHBV production (black arrow). **Figure S2.** Monomer formation in the selected conditions O2, C2 and AL Cells grown in RRNCO medium with fructose 13.3 mM were inoculated at an initial OD_660_ of 0.05, and incubated at 30ºC in constant agitation (200 rpm). The monomer that constituted the polymer was measured along the experiment in O2 (A), C2 (B) and AL (C) conditions. In O2, the curve for 3HB overlaps with the curve for total monomer production (3HB + 3HV). Representative curves of three independent experiments are expressed as mean ± SD. **Figure S3.** *Cell sizes*. The size of more than 100 cells was measured under the optical microscope using Image J software. **Figure S4.** Absorption spectra of cells in 80% glycerol. The near-infrared Qy absorption maximum, corresponding to the carotenoid-containing light harvesting complex (LH1), the three characteristic spirilloxanthin peaks (490 nm, 515 nm, 549 nm) and the absorption maxima of the observable reaction center (RC) at 750 (RC-bound bacteriopheophytin) and 802 nm (accessory bacteriochlorophyll, BChla) were observed in both microaerobic and anaerobic conditions, but not in the aerobic condition as expected. It can be assumed, then, that the PM from C2- and AL-grown cells have a similar composition in terms of pigments, although all the components appreciable in the spectrum are present in higher levels in the condition AL. The spectra were normalised by the average value of the non-pigmented condition (aerobic, O2) so as to emphasise the differences among them. The lighter shadow along each curve, represents the standard deviation of three independent replicates. The absorption peaks corresponding to carotenoids, the BChla (Qx and Qy) and Soret bands (Bx and By) of the LH1 and RC are indicated. **Figure S5.** High amounts of PHBV accumulation in *R. rubrum*. Cells transferred to anaerobic bottles with fresh RRNCO medium (bicarbonate 12.0 mM) accumulating more than 80% CDW PHA, were stained with Nile-red and observed under the optical/fluorescent microscope. The phase contrast image (Top) was merged with the fluorescent image of the same field where the stained granules are shown in red (Bottom). It can be seen that granules occupy most of the cytoplasm.

## Data Availability

Relevant data generated or analysed for this work are included in this published article and its supplementary information.
