# Supplementary material for: Aerobic-anaerobic transition boosts poly(3-hydroxybutyrate-co-3-hydroxyvalerate) synthesis in Rhodospirillum rubrum: the key role of carbon dioxide
Source: Microb Cell Fact. 2023 Mar 10;22:47. doi: 10.1186/s12934-023-02045-x (PMC9999600; doi:10.1186/s12934-023-02045-x)
Supplement: Supplementary file 1 — Additional file 1: Table S1. Summary of the conditions for the open and closed systems. Table S2. Biomass in the late stationary phase and PHBV production and metabolites secreted to the supernatant in the early stationary phase. Table S3. Metabolite production and substrate consumption. Figure S1. Growth curves of R. rubrum in different aeration levels. Open systems O1 (A), O2 (B), O3 (C), and closed systems C1 (D), C2 (E), C3 (F), were grown in darkness, and the anaerobic culture AL (G) was grown with white light. The turbidity (blue curves) is depicted in the same scale as PM production (red curves). Points in the late exponential phase and early stationary phase were taken to assess PHBV production (black arrow). Figure S2. Monomer formation in the selected conditions O2, C2 and AL Cells grown in RRNCO medium with fructose 13.3 mM were inoculated at an initial OD660 of 0.05, and incubated at 30ºC in constant agitation (200 rpm). The monomer that constituted the polymer was measured along the experiment in O2 (A), C2 (B) and AL (C) conditions. In O2, the curve for 3HB overlaps with the curve for total monomer production (3HB + 3HV). Representative curves of three independent experiments are expressed as mean ± SD. Figure S3. Cell sizes. The size of more than 100 cells was measured under the optical microscope using Image J software. Figure S4. Absorption spectra of cells in 80% glycerol. The near-infrared Qy absorption maximum, corresponding to the carotenoid-containing light harvesting complex (LH1), the three characteristic spirilloxanthin peaks (490 nm, 515 nm, 549 nm) and the absorption maxima of the observable reaction center (RC) at 750 (RC-bound bacteriopheophytin) and 802 nm (accessory bacteriochlorophyll, BChla) were observed in both microaerobic and anaerobic conditions, but not in the aerobic condition as expected. It can be assumed, then, that the PM from C2- and AL-grown cells have a similar composition in terms of pigments, although all the componen [file 12934_2023_2045_MOESM1_ESM.pdf]

SUPPLEMENTARY MATERIAL FOR RESEARCH ARTICLE

Aerobic-anaerobic transition boosts poly(3-hydroxybutyrate-co-3-hydroxyvalerate) synthesis in *Rhodospirillum rubrum*: the key role of carbon dioxide.

Manuel S. Godoy [1, 2]*, *Santiago R. de Miguel* [1, 2], *M. Auxiliadora Prieto* [1, 2]*

[1]Polymer Biotechnology Lab, Biological Research Centre Margarita Salas, Spanish National Research Council (CIB-CSIC), Madrid, Spain.
[2]Interdisciplinary Platform for Sustainable Plastics towards a Circular Economy-CSIC (SusPlast-CSIC), Madrid, Spain.
*corresponding authors: auxi@cib.csic.es, msgodoy@cib.csic.es

Table S1. ***Summary of the conditions for the open and closed systems.***

**Condition**	**Agitation** (rpm)	**Filling Vol** (%)	**kLa*** (h-1)	**Darkness (D) or Light (L)**
**O1 / C1**	200	25	49	D
**O2 / C2**	200	50	16	D
**O3 / C3**	100	50	6	D
**AL**	200	50	-	L

(*) The predicted values of kLa (gas–liquid mass transfer coefficient) were estimated considering data published by Schiefelbein *et. al.* [32]. The kLa were calculated fitting an exponential function to their data for 200 rpm or 100 rpm (Shaken frequency) and 125 ml (maximum flask volume). Calculations are valid for disposable non-baffled shake flasks at 30°C and represent only an estimation of the effects of the agitation and the filling volume on the gas transfer rate of our experimental set-up. In the case of closed systems (C1, C2, C3), the kLa would correspond to the initial situation, where the headspace of the bottle is composed of air.

.

Table S2. ***Biomass in the late stationary phase and PHBV production and metabolites secreted to the supernatant in the early stationary phase.***

Condition	Biomass	PHBV			Metabolites in the supernatant (mM)			
	$g \cdot l^{-1}$	$g \cdot l^{-1}$	% CDW	%mol 3HV	Succ	Form	Acet	Prop
O1	0.51 ± 0.07	0.06 ± 0.03	7.0 ± 0.5	14 ± 3	1.2 ± 0.3	0.7 ± 0.1	< 0.05	< 0.1
O2	0.60 ± 0.04	0.04 ± 0.01	4.8 ± 0.2	13 ± 1	1.2 ± 0.4	0.6 ± 0.1	< 0.1	0.2 ± 0.1
O3	0.50 ± 0.00	0.05 ± 0.01	5.0 ± 1.0	17 ± 5	2.2± 0.3	<0.1	2.5± 0.1	1.0 ± 0.2
C1	0.32 ± 0.04	0.03 ± 0.01	3.2 ± 0.2	26 ± 3	1.9 ± 0.4	<0.1	<0.1	1.2 ± 0.6
C2	0.69 ± 0.02	0.20 ± 0.02	29.4 ± 2.3	75 ± 1	3.1 ± 0.7	<0.1	2.3 ± 0.2	3.1 ± 0.1
C3	0.52 ± 0.02	0.24 ± 0.02	31.7 ± 2.2	81 ± 1	2.1 ± 0.1	<0.1	2.0 ± 0.0	2.8 ± 0.3
AL	2.03 ± 0.01	0.04 ± 0.02	4.5 ± 0.6	42 ± 12	1.5 ± 0.1	0.4 ±0.1	2.4 ± 0.6	1.1 ± 0.4

Biomass was measured at the end of the experimental period (6-10 days), while the PHBV and the Metabolites in the supernatant, were measured at the early stationary phase i.e. the time-point of maximal polymer accumulation.

Table S3. ***Metabolite production and substrate consumption.***

	Bic (mM)	Fructose consumption	Acetate	Propionate	Succinate	3HB	3HV	3HB+3HV	CO_2	H_2	Balance (%C)
Concentration (mM)	0	2.5 ± 0.1	1.6 ± 0.2	0.9 ± 0.3	0.6 ± 0.3	0.05 ± 0.02	0.02 ± 0.01	0.06 ± 0.03	1.0 ± 0.1	2.0 ± 2.7	94
	3	15.0 ± 0.2	1.8 ± 0.3	4.2 ± 0.2	7.2 ± 0.7	0.7 ± 0.1	3.9 ± 0.4	4.6 ± 0.5	20.2 ± 1.0	5.3 ± 0.2	88
	6	12.7 ± 1.3	2.0 ± 0.4	4.3 ± 0.2	7.9 ± 0.5	0.7 ± 0.1	4.6 ± 0.4	5.3 ± 0.4	20.4 ± 1.1	5.1 ± 0.5	114
	12	14.9 ± 3.2	2.3 ± 0.2	5.2 ± 0.1	9.1 ± 0.3	0.9 ± 0.1	5.0 ± 0.4	6.0 ± 0.5	25.8 ± 2.3	5.4 ± 0.4	106
Yield ($mol \cdot mol^{-1}$)	0		0.67 ± 0.23	0.34 ± 0.05	0.25 ± 0.17	0.020 ± 0.001	0.01 ± 0.01	0.02 ± 0.01	0.41 ± 0.10	0.16 ± 0.07	
	3		0.12 ± 0.02	0.28 ± 0.02	0.48 ± 0.04	0.050 ± 0.001	0.26 ± 0.03	0.31 ± 0.04	1.35 ± 0.08	0.36 ± 0.01	
	6		0.16 ± 0.02	0.34 ± 0.02	0.62 ± 0.04	0.06 ± 0.01	0.36 ± 0.06	0.42 ± 0.06	1.61 ± 0.10	0.40 ± 0.01	
	12		0.16 ± 0.02	0.36 ± 0.07	0.63 ± 0.12	0.06 ± 0.02	0.35 ± 0.09	0.42 ± 0.11	1.76 ± 0.22	0.37 ± 0.05	
Total gas (mmol)	0								0.010 ± 0.01	0.004 ± 0.003	
	3								0.202 ± 0.01	0.053 ± 0.002	
	6								0.204 ± 0.011	0.051 ± 0.005	
	12								0.258 ± 0.023	0.054 ± 0.004	

Fructose consumption, product formation mM (and mmol in the case of gases) and product yields referred to fructose ($mol \cdot mol^{-1}$) are shown. The mass balance (% C) is also provided.

Fig. S1

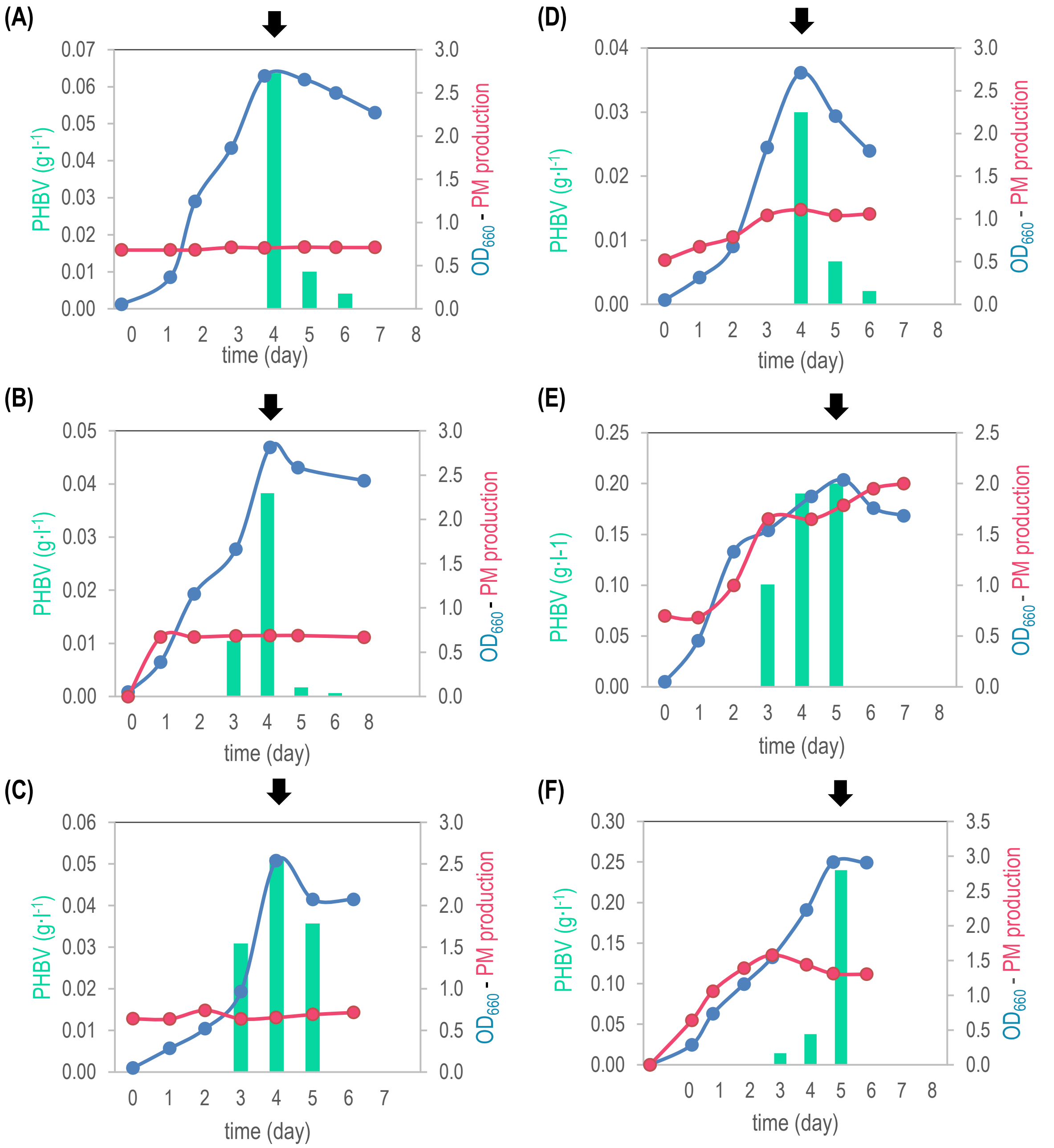

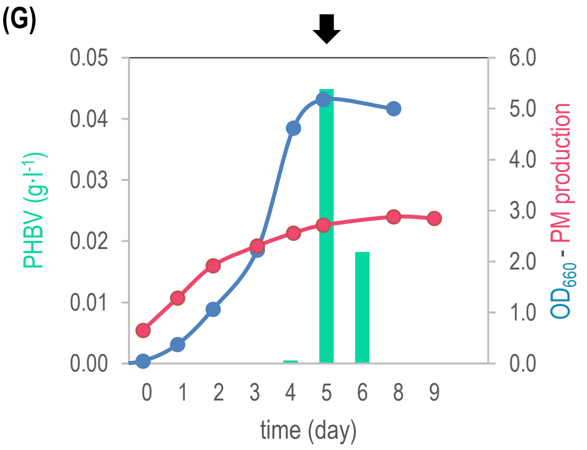

Growth curves of R. rubrum in different aeration levels. Open systems O1 (A), O2 (B), O3 (C), and closed systems C1 (D), C2 (E), C3 (F), were grown in darkness, and the anaerobic culture AL (G) was grown with white light. The turbidity (blue curves) is depicted in the same scale as PM production (red curves). Points in the late exponential phase and early stationary phase were taken to assess PHBV production (black arrow).

Fig. S2

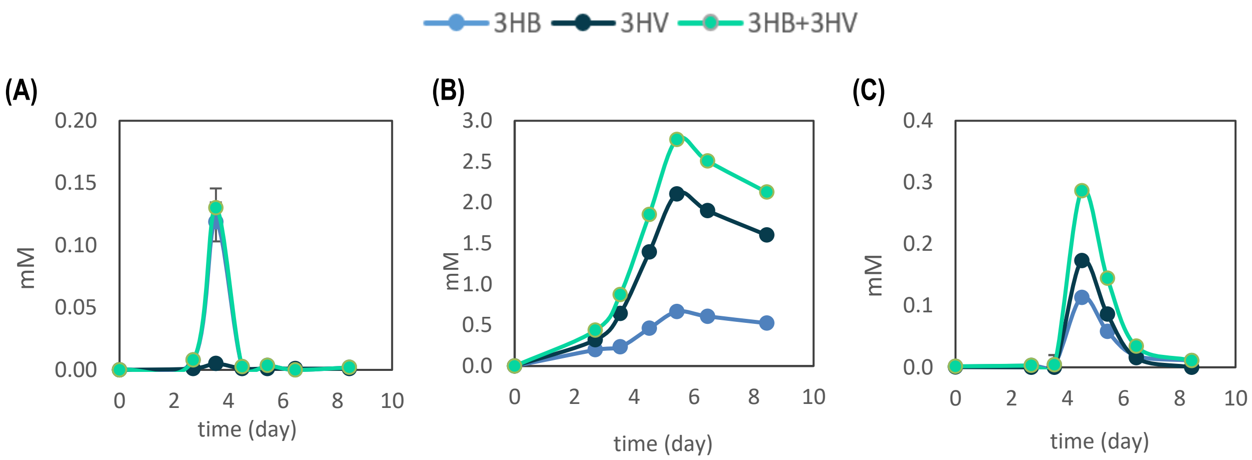

Monomer formation in the selected conditions O2, C2 and AL Cells grown in RRNCO medium with fructose 13.3 mM were inoculated at an initial OD_{660} of 0.05, and incubated at 30ºC in constant agitation (200 rpm). The monomer that constituted the polymer was measured along the experiment in O2 (A), C2 (B) and AL (C) conditions. In O2, the curve for 3HB overlaps with the curve for total monomer production (3HB + 3HV). Representative curves of three independent experiments are expressed as mean ± SD.

Fig S3

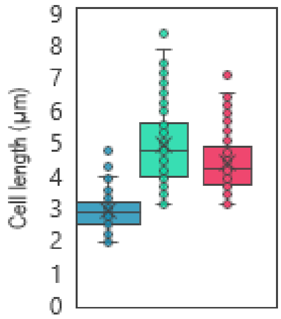

Cell sizes. The size of more than 100 cells was measured under the optical microscope using Image J software.

Fig S4

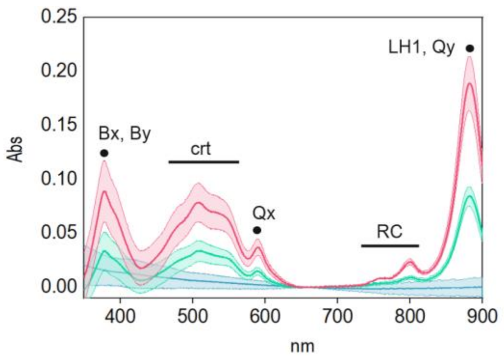

Absorption spectra of cells in 80% glycerol. The near-infrared Qy absorption maximum, corresponding to the carotenoid-containing light harvesting complex (LH1), the three characteristic spirilloxanthin peaks (490 nm, 515 nm, 549 nm) and the absorption maxima of the observable reaction center (RC) at 750 (RC-bound bacteriopheophytin) and 802 nm (accessory bacteriochlorophyll, BChla) were observed in both microaerobic and anaerobic conditions, but not in the aerobic condition as expected. It can be assumed, then, that the PM from C2- and AL-grown cells have a similar composition in terms of pigments, although all the components appreciable in the spectrum are present in higher levels in the condition AL. The spectra were normalised by the average value of the non-pigmented condition (aerobic, O2) so as to emphasise the differences among them. The lighter shadow along each curve, represents the standard deviation of three independent replicates. The absorption peaks corresponding to carotenoids, the BChla (Qx and Qy) and Soret bands (Bx and By) of the LH1 and RC are indicated.

Fig S5

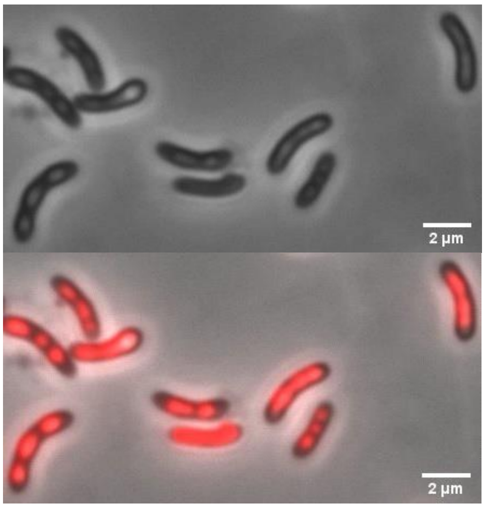

High amounts of PHBV accumulation in *R. rubrum*. Cells transferred to anaerobic bottles with fresh RRNCO medium (bicarbonate 12.0 mM) accumulating more than 80% CDW PHA, were stained with Nile-red and observed under the optical/fluorescent microscope. The phase contrast image (Top) was merged with the fluorescent image of the same field where the stained granules are shown in red (Bottom). It can be seen that granules occupy most of the cytoplasm.
